# Clinical phenotypes of obstructive sleep apnea identified using cluster analysis

**DOI:** 10.36416/1806-3756/e20250497

**Published:** 2026-09-14

**Authors:** Eduardo Borsini, Sebastián Leiva Agüero, Magalí Blanco

**Affiliations:** 1. Servicio de Neumonología. Unidad de Sueño. Hospital Británico de Buenos Aires, Buenos Aires, Argentina.; 2. Unidad de Investigación en Neumonología y Sueño. Fundación Barcelo. La Rioja, Argentina.

**Keywords:** Sleep apnea, obstructive/diagnosis, Symptom assessment, Sleepiness, Cluster analysis

## Abstract

**Objective:**

To describe clinical obstructive sleep apnea (OSA) phenotypes based on emerging groups.

**Methods:**

A cross-sectional study was conducted among adult patients referred for home sleep respiratory assessment. Cluster analysis was conducted based on sex, age, Epworth Sleepiness Scale (ESS) score, BMI, neck > 40 cm (women) or > 42 cm (men), hypertension, and time spent with SpO_2_ below 90% (T<90%).

**Results:**

The K-means algorithm identified three groups: 1) Severe obesity, borderline sleepiness (n = 1,026) with a mean BMI of 34.7 kg/m^2^, ESS score of 9.3, apnea-hypopnea index (AHI) of 31.3 events/h, and T<90% of 4.9 (%); 2) Moderate disease burden, asymptomatic without hypoxemia (n = 497) with a mean BMI = 28.5 kg/m^2^, ESS score = 6.6, AHI = 25.7 events/h, and T<90% of 1.8%; and 3) Sleepy patients with severe OSA (n = 993) with a mean BMI = 29.5 kg/m^2^, ESS score = 13.6, AHI = 33.9 events/h, and T<90% of 3.6%.

**Conclusions:**

In this large sample of Latin American patients with OSA diagnosed using home respiratory polygraphy, cluster analysis identified three clinically relevant phenotypes. Notably, severe OSA was observed both in patients with severe obesity and borderline daytime symptoms and in overweight patients with excessive daytime sleepiness. These findings highlight the heterogeneity of OSA and emphasize the need for individualized phenotype-based clinical approaches.

## INTRODUCTION

Obstructive sleep apnea (OSA) is a prevalent disease with a significant impact on public health.^1^ OSA is a multifactorial condition with a heterogeneous clinical presentation affecting over 1 billion people worldwide.^2^ In Latin America, approximately 10% of women and at least 20% of men in the adult general population suffer from OSA, with concerning rates of underdiagnosis.^3^

It has been suggested that OSA is characterized by distinct underlying mechanisms (endotypes) and varying clinical presentations (clinical phenotypes) based on patients’ dominant pathophysiological mechanisms,^4^ ^-^ ^6^ and associated comorbidities, among other factors.^5^ ^,^ ^6^

From a pragmatic perspective, endotyping requires a comprehensive semiological analysis of polysomnography results^6^ and supplementary tests (e.g. sleep center-specific CPAP titration protocols and esophageal pressure monitoring).^7^ However, in regions where sleep centers are scarce or poorly equipped,^8^ however, diagnoses are made using home sleep tests.^9^

Strategies to estimate OSA severity and identify different types of clinical presentations include multiple variables, such as age, BMI, nocturnal desaturation, apnea-hypopnea index (AHI), daytime symptoms measured by the Epworth Sleepiness Scale (ESS), and cardiovascular comorbidities.^1^ Therefore, identifying the clinical patterns and forms of presentation of OSA in our population is particularly relevant. 

Furthermore, recent data underscore the importance of oximetry findings in OSA patients due to their association with mortality rates^10^ and cardiovascular prognosis,^11^ regardless of the AHI obtained from conventional sleep studies.^12^ Oximetry findings can be quantified using metrics from simplified sleep tests,^11^ ^,^ ^12^ which can be useful to stratify patients for decision-making purposes. 

Thus, the purpose of this study was to identify clinical phenotypes of OSA in patients referred to the sleep unit of a general hospital for home respiratory polygraphy, using cluster analysis to identify data-driven groups that incorporate oximetry findings.

## METHODS

This was a single-center, cross-sectional study based on a systematic database search of adult patients referred for self-administered home respiratory polygraphy (RP) at a community hospital. 

The protocol was approved by the local independent ethics committee and the national regulatory authority (PRIISA.BA), and the study was conducted in accordance with the principles of the Declaration of Helsinki, as amended for medical research involving human subjects (Protocol No. 20210-2025). 

We included patients aged ≥ 18 years who underwent home respiratory polygraphy between 2013 and 2019 (6-year period). Recordings with significant artifacts in respiratory or oximetry signals were excluded, as were recordings with a total recording time (TRT) < 240 min, and patients receiving CPAP or supplemental oxygen therapy. Figure 1 shows the flow diagram of patient selection.

**Figure 1:**
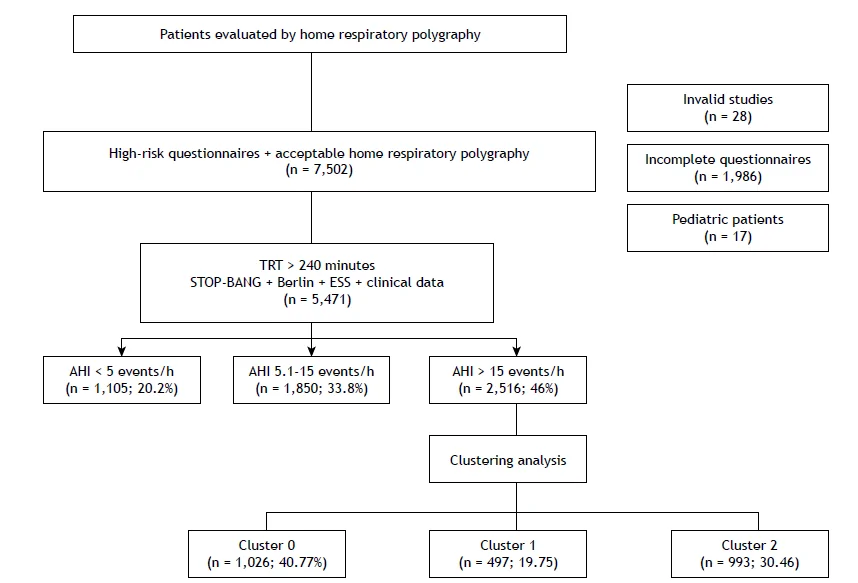
Flow chart of patient selection. TRT: total recording time; ESS: Epworth Sleepiness Scale; and AHI: apnea-hypopnea index.

Home respiratory polygraphy was prescribed and interpreted by trained sleep medicine physicians. Patients used ApneaLink (ResMed, Australia) devices.^9^ These Level III devices measure airflow and snoring via a nasal cannula, respiratory effort through a thoracoabdominal belt, and pulse oximetry.^9^ Following automated signal analysis using ApneaLink software version 10.2, respiratory events were manually reviewed and corrected by sleep medicine-trained physicians.^9^

Apnea was defined as a reduction of airflow ≥ 90% for ≥ 10 seconds, and hypopnea as either a ≥ 50% reduction in the airflow signal or an identifiable airflow reduction accompanied by a ≥ 3% decrease in oxygen saturation for ≥ 10 seconds in either case.^1^ ^,^ ^13^ The oxygen desaturation index (ODI) expressed as events per hour of recording was calculated from oxygen desaturation ≥ 3%, excluding artifact periods.

The AHI was defined as the number of respiratory events (apneas + hypopneas) per valid total recording time, expressed as events/h. OSA was classified as mild (AHI between 5.0 and 14.9 events/h), moderate (AHI between 15.0 and 29.9 events/h), or severe (AHI > 30.0 events/h). Clinically relevant OSA was defined as an AHI ≥ 15.0 events/h.^1^

All patients completed Spanish-language versions of the STOP-BANG Questionnaire (SBQ),^14^ Berlin Questionnaire (BQ),^15^ and ESS.^16^ Excessive daytime sleepiness (EDS) was defined as an ESS score > 10. Depending on the number of BQ categories, pretest probability was classified as high (≥ 2 positive categories) or low (just 1 positive category).

Patients were considered to have systemic arterial hypertension when high blood pressure was documented in their clinical record or if they were on antihypertensive medication.^17^ Overweight was defined as a BMI between 25 and < 30 kg/m^2^, and obesity as a BMI ≥ 30 kg/m^2^.

Eligible patients (AHI > 15 events/h) underwent descriptive and cluster analyses (unsupervised clustering) to identify emerging phenotypes based on sex, age, ESS score, BMI (kg/m^2^), neck circumference > 40 cm (women) or > 42 cm (men), arterial systemic hypertension and T<90%. This approach involved adapting the model for categorical variables and segmented the population without pre-established clinical definitions, relying exclusively on statistical patterns emerging from available data.

Variable distribution was assessed using frequency histograms and the Kolmogorov-Smirnov test. Values were expressed either as means and standard deviations or medians and interquartile ranges. Group comparisons were performed using parametric or nonparametric tests according to data distribution. The Kruskal-Wallis test was used for quantitative variables, and the chi-square test was used for categorical values. 

The variables age, ESS, ODI (3% criterion), BMI, and T<90% were standardized by principal component analysis, a dimensionality-reduction technique. 

K-means clustering was then conducted to identify alternative phenotypes and assign each patient to a specific group. Given that the analyzed clinical variables were measured on different units and scales, all continuous variables were previously standardized (Z-score transformation) before clustering to ensure equal weighting in distance calculations. Squared Euclidean distance was used as the similarity measure. The optimal number of clusters (k) was determined through multiple iterations using the elbow method and Silhouette score analysis, which indicated that a three-cluster solution provided the best clinical and statistical separation.

Statistical calculations were carried out with MedCalc version 20.027 (MedCalc Software Ltd, Ostend, Belgium), Prism 7 (Graph Pad Software, La Jolla, CA. USA) and IBM SPSS Statistics version 30 (IBM Corp., Armonk, NY, USA).

## RESULTS

A total of 7,502 respiratory polygraphy recordings were obtained over a 6-year period. Of these, 2,516 (46%) had an AHI > 15 events/h and constituted our population of interest. The final cohort was predominantly male (n = 1,897; 75.4%), with a median age of 58 (48-67) years, BMI of 32.0 (28.7-37.0) kg/m^2^ (67% were considered obese), ESS score of 7 (4-12), and a high-risk Berlin score in 93.7% of participants.

Women were underrepresented, were older, and had a higher proportion of obesity. Systemic arterial hypertension was diagnosed in 60% of participants. Table 1 shows the characteristics of the sample population. 

**Table 1:** Characteristics of the study population (N = 2,516).

Clinical characteristics	Total sample	Female (n = 619) (	Male (n = 1,897)	p*
Patient, %	100.0	24.6	75.4	-
Age, years	58 (48-67)	60 (51-69)	57 (47-66)	< 0.001
BMI, kg/m^2^	32 (28.7-37.0)	34 (29.0-40.1)	32 (28.4-36.0)	0.001
BMI ≥ 30 kg/m^2a^	1,689 (67.1)	446 (72.0)	1,243 (65.5)	0.003
Neck circumference^a,b^	1,780 (70.7)	355 (57.3)	1,425 (75.1)	< 0.001
ESS score	7 (4-12)	7 (4-12)	8 (4-12)	0.040
ESS > 10^a^	788 (31.3)	182 (29.4)	606 (31.9)	0.250
High-risk Berlin questionnaire^a^	2,358 (93.7)	578 (93.3)	1,780 (93.8)	0.700
High blood pressure^a^	1,530 (60.8)	370 (59.7)	1,160 (61.1)	0.419
Polygraphy indicators				
AHI, events/h	28 (20-41)	24 (19.0-34.6)	29 (21-43)	0.001
ODI, events/h	28 (21-49.3)	25.3 (20-35.5)	29.9 (21.4-43.0)	0.001
T<90%, absolute %	17 (7-39)	17 (7-39)	18 (7-38)	0.640
T<90% > 3% TRT^a^	2,189 (87.0)	527 (85.1)	1,662 (87.6)	0.113
T<90% > 10% TRT^a^	1,647 (65.5)	369 (59.6)	1,278 (67.3)	0.001
T<90% > 30% TRT^a^	839 (33.3)	214 (34.5)	625 (32.9)	0.461

ESS: Epworth Sleepiness Scale. AHI: apnea-hypopnea index. ODI: oxygen desaturation index. T<90%: oxygen saturation < 90% during the TRT (total recording time) valid for the analysis. Data presented as median (IQR), except where otherwise indicated. ^a^Number and proportion of patients, n (%). ^b^Neck circumference > 42 cm in males and > 40 cm in females. *Kruskal Wallis test or chi-square test for independent variables.

An ESS score > 10 points was found in one-third of the population, with no significant differences between men and women.

Figure 2 shows the correlation between the severity of nocturnal hypoxemia and increasing AHI (Figure 2A), age (Figure 2B), and BMI (Figure 2C). 

**Figure 2:**
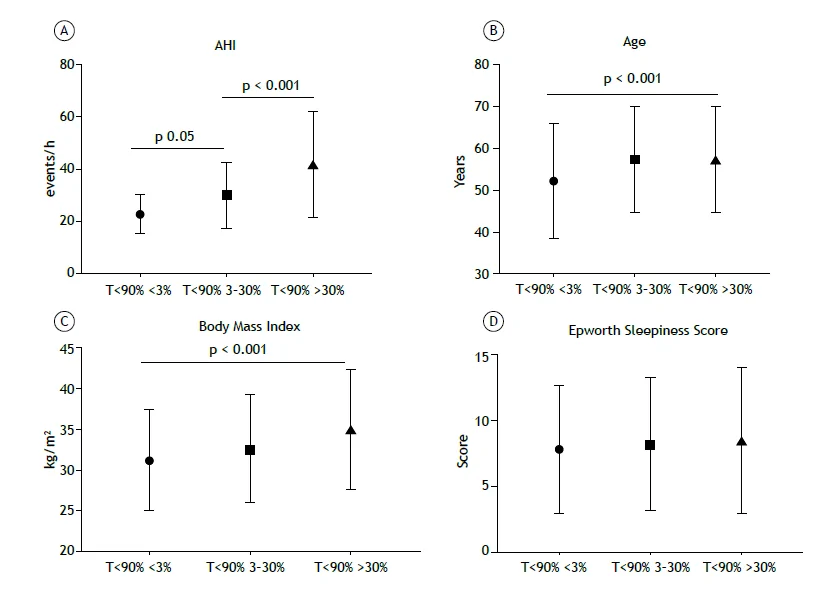
Correlation between the severity of nocturnal hypoxemia and the increase in apnea-hypopnea index (in A), age (in B), Body Mass Index (in C) and Epworth Sleepiness Scale (in D).

The K-means algorithm identified three clusters. Table 2 presents the resulting centroids and emerging clusters. Three distinct phenotypes were found:

**Table 2:** Analysis of main components, dimensional reduction, and processing using the K-means algorithm to identify emerging groups (clusters).

Selected variable	Cluster 0	Cluster 1	Cluster 2
Patients, n (%)	1,026 (40.77)	497 (19.75)	993 (39.46)
Age, years	55.77	51.15	55.02
ESS score	9.27	6.55	13.55
AHI, events/h	31.34	25.68	33.93
ODI, events/h	26.34	16.94	30.17
BMI, kg/m^2^	34.69	28.49	29.49
T<90%, % of TTR	4.91	1.83	3.61

ESS: Epworth Sleepiness Scale. AHI: apnea-hypopnea index. ODI: oxygen desaturation index. T<90%: oxygen saturation < 90% during the TRT (total recording time) valid for the analysis.

Data are presented as centroid, except where otherwise indicated

Cluster 0: “Severe obesity, borderline sleepiness” (n = 1,026; 40.8%), characterized by middle-aged patients (mean age ~ 55 years) with severe obesity (mean BMI = 34.7 kg/m^2^), borderline daytime sleepiness (ESS ~ 9.3 points), and a high burden of respiratory events (mean AHI = 31.3 events/h; mean ODI 26.3 events/h), despite a low hypoxia level (T<90% = 4.9%).

Cluster 1: “Moderate disease burden, asymptomatic without hypoxemia” (n = 497; 19.8%), characterized by slightly younger patients (mean age ~ 51 years) who are overweight (mean BMI = 28.5 kg/m^2^), with normal ESS level (< 7 points), moderate OSA (mean AHI = 25.7 events/h), and minimal nocturnal hypoxemia (T<90% = 1.8%).

Cluster 2: “Sleepy patients with severe OSA” (n = 993; 39.46%) characterized by middle-aged patients (mean age ~ 55 years) who were overweight (mean BMI = 29.5 kg/m^2^) and presented with excessive daytime sleepiness (ESS > 13) and a severe burden of respiratory events (AHI = 33.9 events/h; ODI = 30.1 events/h).

Table 3 summarizes the characteristics of the three groups identified by the cluster analysis. 

**Table 3:** Description of emerging groups identified using cluster analysis.

Variable	Cluster 0 (n = 1,026)	Cluster 1 (n = 497)	Cluster 2 (n = 993)
	Severe obesity, borderline sleepiness	Moderate disease burden, asymptomatic without hypoxemia	Sleepy patients with severe OSA
Age (years)	Middle-aged (50-60)	Middle-aged (45-55)	Middle-aged (50-60)
ESS (score)	Borderline (8-10)	Low (< 8)	High (> 11)
AHI (events/h)	Severe (> 30)	Moderate (20-30)	Severe (> 30)
ODI (events/h)	Moderate (20-30)	Mild to Moderate (15-20)	Severe (> 30)
BMI (kg/m^2^)	Severe obesity (> 34)	Overweight-Mild obesity (25-30)	Overweight-Mild Obesity (25-30)
T<90% (% of TTR)	Low (< 5)	High (> 25)	Intermediate (10-30)

ESS: Epworth Sleepiness Scale. AHI: apnea-hypopnea index. ODI: oxygen desaturation index. T<90%: oxygen saturation < 90% during the TRT (total recording time) valid for the analysis.

## DISCUSSION

This study, based on a large sample of adult OSA patients, identified three phenotypes according to their clinical, anthropometric, and functional characteristics. The analyzed cohort consisted predominantly of men at a high risk for OSA, based on administered questionnaires, representing the typical population referred for this type of home sleep test. Women were underrepresented, as is often the case in OSA studies.^18^

Our cluster analysis identified different phenotypes of OSA patients by segmenting the population without predefined assumptions (fuzzy limits), relying only on emerging statistical patterns from available data. The K-means algorithm does not establish explicit inclusion criteria; rather, it infers patterns from available data, whereas supervised classification methods use rigid or fixed clinical rules or exclusion criteria. This approach groups patients by inferring approximate criteria, allowing some degree of uncertainty, and identifies the central values or ranges (centroid and standard deviation) of each cluster. These clusters characteristics can be used as reference thresholds for classifying new patients or describing the shared characteristics of each phenotype. Thus, we were able to identify three mutually exclusive and clinically relevant phenotypes that are not recognized by conventional classifications.^1^

The first group, accounting for the largest proportion of our patients (Cluster 0; > 40%), was characterized as “Severe obesity with borderline sleepiness.” Despite their extreme obesity and high AHI, they do not present with frank excessive daytime sleepiness (mean ESS ~ 9). This phenotype is clinically highly relevant, as it highlights the risk of underestimating OSA if clinical screening relies too heavily on the ESS, emphasizing the need for objective measurements in obese patients.

The second group (Cluster 1: ~ 20%) comprised patients with “Moderate disease burden, asymptomatic without hypoxemia.” These individuals are typically younger, overweight rather than obese, and reported few daytime symptoms. In clinical practice, they are often referred due to witnessed apneas or snoring rather than self-reported symptoms. Because exhibit minimal daytime symptoms and without significant hypoxemia, these patients might perceive to be at a lower cardiovascular risk and may delay seeking medical care or show poor adherence to CPAP therapy, requiring targeted education.

The third group (Cluster 2: ~ 40%) represented the “Sleepy patients with severe OSA”. Interestingly, despite having a lower BMI (overweight or mild obesity) than patients in Cluster 0, they exhibited the highest ESS scores and the greatest burden of respiratory events. This phenotype underscores that severe sleep fragmentation and daytime hypersomnolence may occur independently of extreme obesity, likely reflecting different anatomic or functional traits (endotypes). While their major complaint is typically tiredness or excessive somnolence, these patients represent the classic high-risk OSA phenotype requiring prompt therapeutic intervention.

Unlike the cohort studies conducted by Keenan et al.,^19^, Trzepizur et al.,^20^ and Mazzotti et al.,^21^ which found four subgroups using cluster analysis, our study only found three. Other authors have described a subgroup of patients with “disturbed sleep,”^19^ ^-^ ^21^ which could not be identified in our cohort because data on depression and insomnia were unavailable. Some of these patients may have been included within the “Moderate disease burden, asymptomatic without hypoxemia” phenotype.

In OSA patients, hypoxemia is an independent predictor of cardiovascular events and death. Some studies suggest that symptom-based subtypes have lower predictive value with regard to complications.^20^ ^,^ ^22^ Likewise, our group classification approach links symptoms (standardized questionnaires) to easily assessed anthropometric characteristics (neck circumference and BMI) and objective findings from respiratory polygraphy, including indicators and hypoxemia patterns. This strategy may be useful for prioritizing waiting lists, improving risk, and tailoring treatment or therapeutic strategies.^19^ ^,^ ^22^

This study has several limitations. First, it was a retrospective study. Although the systematic collection of data was standardized, it suffers from all the typical biases of this type of design. Second, since our patients were preselected based on a suspicion of OSA, our results may not be generalizable to other populations. Third, we used a “strict” criterion for the AHI (> 15 events/h) based on home respiratory polygraphy,^1^ even though these tests are known to underestimate AHI.^1^ ^,^ ^13^ Fourth, we failed to include other comorbidities (other than hypertension) that could have otherwise affected our way of grouping patients.^22^ Fifth, the metrics we use to assess hypoxemia did not include the calculation of hypoxic burden. Sixth, although clustering segmentation has technical limitations, our analysis included a large sample with few extreme outliers, supporting the robustness of the identified clusters. Finally, the identified phenotypes were not linked to clinical outcomes, such as cardiovascular events, mortality, or response to continuous positive airway pressure therapy. Without prospective or functional validation, the clusters identified here remain descriptive, a limitation present in previous studies, and this substantially reduces the potential impact of our data.

In conclusion, in this extensive sample of Latin American patients with OSA diagnosed using home respiratory polygraphy, cluster analysis identified three clinically relevant phenotypes. Notably, severe OSA was observed both in patients with severe obesity and borderline daytime sleepiness and in overweight patients with excessive daytime somnolence, demonstrating that classical symptoms do not always parallel obesity or AHI. These data-driven findings highlight the heterogeneity of OSA and emphasize the need for individualized, phenotype-based clinical approaches.

## References

[1] Mediano O, González Mangado N, Montserrat JM, Alonso-Álvarez ML, Almendros I, Alonso-Fernández A (2022). International Consensus Document on Obstructive Sleep Apnea. Arch Bronconeumol.

[2] Benjafield AV, Ayas NT, Eastwood PR, Heinzer R, Ip MSM, Morrell MJ (2019). Estimation of the global prevalence and burden of obstructive sleep apnoea a literature-based analysis. Lancet Respir Med.

[3] Tufik S, Santos-Silva R, Taddei JA, Bittencourt LR (2010). Obstructive sleep apnea syndrome in the Sao Paulo Epidemiologic Sleep Study. Sleep Med.

[4] Ye L, Pien GW, Ratcliffe SJ, Björnsdottir E, Arnardottir ES, Pack AI (2014). The different clinical faces of obstructive sleep apnoea a cluster analysis. Eur Respir J.

[5] Malhotra A, Mesarwi O, Pepin JL, Owens RL (2020). Endotypes and phenotypes in obstructive sleep apnea. Curr Opin Pulm Med.

[6] Eckert DJ, White DP, Jordan AS, Malhotra A, Wellman A (2013). Defining phenotypic causes of obstructive sleep apnea Identification of novel therapeutic targets. Am J Respir Crit Care Med.

[7] Edwards BA, Landry SA, Thomson LDJ, Joosten SA (2025). Sleep apnea endotypes and their implications for clinical practice. Sleep Med.

[8] Lorena Noriega-Aguirre L, Franceschini CM, Mercedes Alonzo-de Rodríguez A, Arias-Arias PA, Blanquicett-Barrios L, Byrne JP (2024). Coverage for the Diagnosis and Treatment of Obstructive Sleep Apnea in Latin America: Report from the Latin American Forum of Respiratory Societies [Article in Spanish]. Respirar.

[9] Borsini E, Blanco M, Bosio M, Fernando DT, Ernst G, Salvado A (2016). Diagnosis of sleep apnea in network Respiratory polygraphy as a decentralization strategy. Sleep Sci.

[10] Azarbarzin A, Sands SA, Stone KL, Taranto-Montemurro L, Messineo L, Terrill PI (2019). The hypoxic burden of sleep apnoea predicts cardiovascular disease-related mortality the Osteoporotic Fractures in Men Study and the Sleep Heart Health Study. Eur Heart J.

[11] Blekic N, Bold I, Mettay T, Bruyneel M (2022). Impact of Desaturation Patterns versus Apnea-Hypopnea Index in the Development of Cardiovascular Comorbidities in Obstructive Sleep Apnea Patients. Nat Sci Sleep.

[12] Malhotra A, Ayappa I, Ayas N, Collop N, Kirsch D, Mcardle N (2021). Metrics of sleep apnea severity beyond the apnea-hypopnea index. Sleep.

[13] Berry RB, Budhiraja R, Gottlieb DJ, Gozal D, Iber C, Kapur VK (2012). American Academy of Sleep Medicine Rules for scoring respiratory events in sleep: update of the 2007 AASM Manual for the Scoring of Sleep and Associated Events. Deliberations of the Sleep Apnea Definitions Task Force of the American Academy of Sleep Medicine. J Clin Sleep Med.

[14] Chung F, Abdullah HR, Liao P (2016). STOP-Bang Questionnaire A practical approach to screen for obstructive sleep apnea. Chest.

[15] Netzer NC, Stoohs RA, Netzer CM, Clark K, Strohl KP (1999). Using the Berlin Questionnaire to identify patients at risk for the sleep apnea syndrome. Ann Intern Med.

[16] Chiner E, Arriero JM, Signes-Costa J, Marco J, Fuentes I (1999). Validation of the Spanish version of the Epworth Sleepiness Scale in patients with a sleep apnea syndrome. Arch Bronconeumol.

[17] Borsini E, Nigro C (2023). Hypoxemia and hypertension in obstructive sleep apnea the forgotten variable. J Bras Pneumol.

[18] Nigro CA, Dibur E, Borsini E, Malnis S, Ernst G, Bledel I (2018). The influence of gender on symptoms associated with obstructive sleep apnea. Sleep Breath.

[19] Keenan BT, Kim J, Singh B, Bittencourt L, Chen NH, Cistulli PA (2018). Recognizable clinical subtypes of obstructive sleep apnea across international sleep centers a cluster analysis. Sleep (Basel).

[20] Trzepizur W, Blanchard M, Ganem T, Balusson F, Feuilloy M, Girault JM (2022). Sleep Apnea-Specific Hypoxic Burden, Symptom Subtypes, and Risk of Cardiovascular Events and All-Cause Mortality. Am J Respir Crit Care Med.

[21] Mazzotti DR, Keenan BT, Lim DC, Gottlieb DJ, Kim J, Pack AI (2019). Symptom subtypes of obstructive sleep apnea predict incidence of cardiovascular outcomes. Am J Respir Crit Care Med.

[22] Sánchez-de-la-Torre M, Sánchez-de-la-Torre A, Bertran S, Abad J, Duran-Cantolla J, Cabriada V (2020). Spanish Sleep Network Effect of obstructive sleep apnoea and its treatment with continuous positive airway pressure on the prevalence of cardiovascular events in patients with acute coronary syndrome (ISAACC study): a randomised controlled trial. Lancet Respir Med.

